# Proton-Sensitive Receptors/Channels: Therapeutic Target for Ischemic Stroke, Focusing on Their Roles and Mechanisms

**DOI:** 10.2174/011570159X381737250710050221

**Published:** 2025-07-18

**Authors:** Yao Cheng, Yu-Jie Zhai, Shao-Shuai Wang, Yan-Ying Fan

**Affiliations:** 1 Department of Pharmacology, School of Basic Medical Science, Shanxi Medical University, Taiyuan, Shanxi, 030001, China;; 2 Medicinal Basic Research Innovation Center of Chronic Kidney Disease, Ministry of Education, Shanxi Medical University, Taiyuan, 030001, China;; 3 Department of Neurology, First Hospital of Shanxi Medical University, Taiyuan, 030001, China;; 4 Key Laboratory of Cellular Physiology, Ministry of Education, Shanxi Medical University, Jinzhong, 030600, China

**Keywords:** Ischemic stroke, acidosis, proton-sensitive receptors/channels, neuroprotection, neurorestoration, neuroinflammation, therapeutic targets

## Abstract

Ischemic stroke is a common cerebrovascular disease. However, its pathophysiological mechanisms and therapeutic targets remain unclear. In physiological states, the brain maintains an acid-base balance through various mechanisms that are crucial for normal brain function. However, during cerebral ischemia, the disruption of this balance leads to acidosis of the ischemic brain tissue, primarily due to the interruption of energy supply and alterations in ion homeostasis. Studies have indicated that proton-sensitive receptors/channels, such as acid-sensing ion channels, proton-sensitive G-protein-coupled receptors, and proton-activated chloride channels, exhibit distinct mechanisms in response to decreased pH. These targets are intricately linked to the pathophysiological processes, such as neuroprotection, neurorestoration, and neuroinflammation, underlying ischemic stroke. Recent studies have uncovered novel patterns of recognition, activation, subcellular distribution, and emerging functions of these proton-sensing receptors/channels, offering deeper insights into their roles and mechanisms in ischemic brain injury. This review summarizes the current insights regarding different contributions of these proton-sensing receptors/channels following ischemic stroke, and highlights the significance of targeting them for advancing novel therapeutic strategies for stroke intervention.

## 1 INTRODUCTION

 Ischemic stroke, a major cause of disability and mortality, is one of the most prevalent cerebrovascular diseases, resulting from reduced blood supply to a specific brain region due to vascular occlusion [1, 2]. A series of studies have confirmed that cerebral acid-base balance is dynamically perturbed following ischemic stroke [3, 4]. Physiologically, the brain contains massive bicarbonate ion buffering pairs that maintain cellular pH within the range of 7.2-7.4 [5]. When cerebral ischemia occurs, the pH of the ischemic core rapidly decreases to ~6.0, while that of the peri-infarct penumbra decreases to 6.5~6.9 [6]. The severe acidosis leads to metabolic energy disorders, accelerates the oxyradical production, exacerbates blood-brain barrier disruption, and promotes neuronal death [6-8], all of which are acknowledged as pathological progresses in cerebral ischemia. Conversely, some studies have indicated that transient mild acidosis in brain tissue may confer neuroprotective effects against ischemic brain injury [9-11]. For example, CO_2_ inhalation within minutes after cerebral ischemia/reperfusion produces neuroprotection *via* suppressing the opening of the mitochondrial permeability transition pore and augmenting PARK-mediated mitophagy [9, 11]. These findings have promoted further explorations into the mechanisms and therapeutic targets of acidosis in ischemic stroke.

 In the past few decades, several proton-sensitive receptors/channels have been identified, which can be recognized and interacted with directly by protons, including proton-sensitive G Protein-Coupled Receptors (GPCRs), Acid-Sensing Ion Channels (ASICs), and Proton-Activated Chloride channels (PACs) [5, 12]. These receptors/channels are critically involved in the pathophysiological progression of ischemic stroke. Recently, Zha *et al*. summarized the functional heterogeneity of proton-sensitive receptors/channels, highlighting their various contributions to mechanisms in the acute phase of cerebral ischemic injury [5]. Since then, many novel works on these receptors/channels in the context of cerebral ischemia have been published. For instance, recent studies have demonstrated that certain proton-sensitive receptors, such as T cell death-associated gene 8 (TDAG8), exert neuroreparative effects in the subacute phase following cerebral ischemia [13, 14]. Meanwhile, the downstream signaling pathways of ovarian cancer G protein-coupled receptor 1 (OGR1) mediated neuroprotection have been further explored [15]. In addition, Lai *et al.* have found that glutamate exacerbates ischemic brain injury through the positive allosteric regulation of ASIC1a [16]. 

**Fig. (1):**
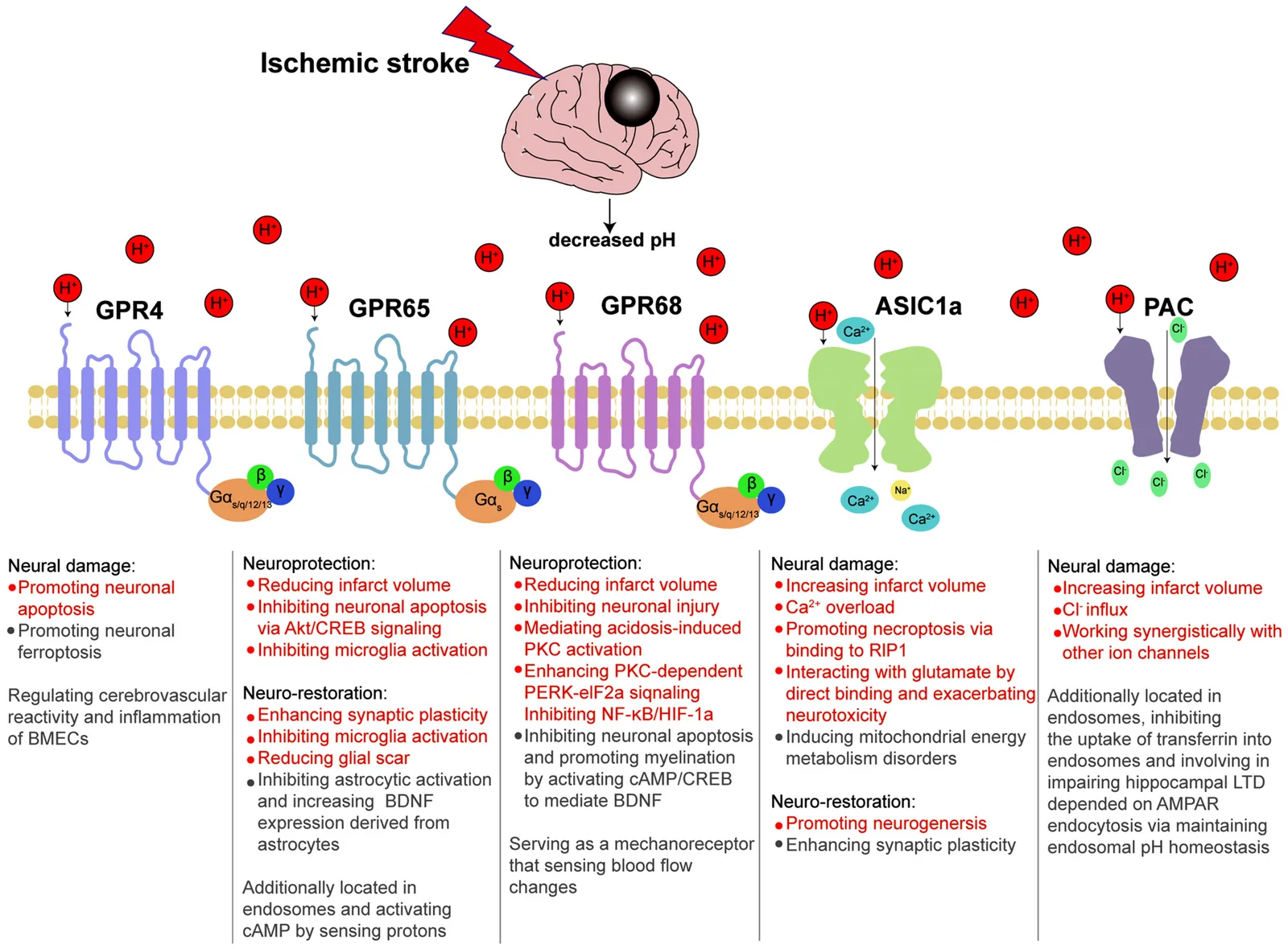
The effects of proton-sensitive receptors/channels in ischemic stroke. The schematic representation shows distinct roles of GPR4, TDAG8, OGR1, ASIC1a, and PAC. Red text: validated in the *in vivo* or *in vitro* model of cerebral ischemia; gray text: not validated in the *in vivo* or *in vitro* model of cerebral ischemia. BDNF, brain-derived neurotrophic factor; BMECs, brain microvascular endothelial cells; CREB, cAMP-responsive element-binding protein; PKC, Protein Kinase C; RIP1, intracellular death-inducing kinase.

Therefore, this review systematically consolidated the roles, characteristics, and mechanisms of proton-sensitive receptors/channels under cerebral ischemic conditions from pioneering discoveries to the most recent advances (Fig. **1**). Additionally, the current drugs targeting them and intervention strategies are also summarized, highlighting the clinical translational potential of these targets.

## 2 SEARCHING METHODOLOGY

 In this review, PubMed was searched for original articles and reviews related to ‘proton sensitive receptors/channels in ischemic stroke’ in English from 2005 to 2025. The following searching keywords were used: (Proton-sensitive receptors) AND (Ischemic stroke OR Cerebral ischemia); (GPR4) OR (TDAG8) OR (OGR1) OR (ASIC1a) OR (PAC). The following studies were excluded: (1) studies irrelevant to the theme of “proton sensitive receptors/channels in ischemic stroke”; (2) studies exhibiting wrong data or parameters; (3) studies that were not available or retracted. The main studies on proton-sensitive receptors/channels in the context of cerebral ischemia in the last ten years are presented in Table **1** [13, 15-27].

## 3 PROTON-SENSITIVE GPCRS

 Proton-sensing GPCRs are a subclass of GPCRs that detect changes in extracellular pH and initiate intracellular signal transduction. To date, four GPR family members have been identified, including OGR1, TDAG8, G protein receptor 4 (GPR4), and GPR132. Compared to ASICs and PAC, they exhibit higher sensitivity to changes in brain pH [5]. This sensitivity is attributed to critical histidine residues whose imidazole groups possess protonation capabilities. When the extracellular acidity increases, protons bind to these imidazole groups, destabilizing the hydrogen bonds between histidines [28]. This structural change may activate receptors, triggering their interaction with G proteins and further activating downstream signaling pathways. Generally, GPR4, TDAG8, and OGR1 couple to G_s_, while GPR4 and OGR1 can also couple to G_q_ and G_12/13_ [28, 29]. Among these receptors in humans, GPR4 is activated at pH 7.6-8.4, TDAG8 is activated at pH 6.8-7.4, and OGR1 is activated at pH 6.0-7.0 [30]. Although GPR132 responds to pH changes, its pH sensitivity is much weaker than that of the other three members [31]. Structural analysis revealed that the conserved proton-sensing amino acid residues of the other three proton-sensing GPCRs limited pH-dependent signal transduction [32]. Additionally, RNA sequencing revealed that GPR132 was not expressed in human and mouse brains compared to the other three members. In this section, the focus is on OGR1, TDAG8, and GPR4.

### 3.1 OGR1

 OGR1 was originally found in a human ovarian cancer cell line. Hence, it was named “ovarian cancer G protein-coupled Receptor 1” [33]. OGR1 is considered a mechanoreceptor activated by cellular stretch and acidosis, and it exhibits minimal rapid desensitization after its activation. One study suggested that OGR1 is present in cerebellar granulosa cells and contributes to calcium signaling. However, recent studies have found that OGR1 exhibits a widespread pattern of expression across brain neurons, with small amounts present in endothelial and immune cells, as determined through *in vitro* and *in vivo* immunostaining experiments [19, 34].

#### 3.1.1 The Neuroprotective Roles of OGR1 in Ischemic Stroke

Recent studies have shown that OGR1 has strong neuroprotective properties against neuronal injury [15, 17, 35]. In the sevoflurane-induced cerebral injury model in rats, OGR1 overexpression mediated the secretion of brain-derived neurotrophic factor (BDNF) *via* increasing cyclic adenosine monophosphate (cAMP) and phosphorylated cAMP-responsive element-binding protein (CREB), then alleviated sevoflurane-induced neuronal apoptosis and demyelination, and improved learning and memory function in rats [35]. Recently, a series of studies has demonstrated that neuronal OGR1 activation in response to cerebral ischemia-induced acidosis produced robust neuroprotective effects in a transient middle cerebral artery occlusion/refusion (tMCAO) mouse model [15, 19]. Using the bacterial artificial chromosome BAC Gpr68-eGFP reporter mouse, Wang *et al*. found that OGR1 expressed in both the cerebral cortex and striatum colocalizes with neurons [19]. Meanwhile, OGR1 deletion increased cerebral infarction and neurological deficits in mice 24 and 72 h after reperfusion, whereas overexpressing OGR1 reduced cerebral infarction. Additionally, this study revealed that the neuroprotective effect of OGR1 was dependent on the activation of Protein Kinase C (PKC). This enzyme plays a crucial role in cell survival and apoptosis. Go6983 (a selective PKC inhibitor) treatment worsened acid-induced injury in wild-type but not OGR1 knockout brains. Furthermore, they confirmed that OGR1 activation through intravenous injection of Ogerin (a positive modulator of OGR1) at 3 and 5 h post-reperfusion alleviated the volume of infarction and decreased the expressions of inflammatory factors, and that OGR1 knockout reversed these beneficial effects [15]. Recently, Zhou *et al.* explored transcriptome changes and downstream signaling mediated by OGR1 in acute cerebral ischemia *via* performing RNA-seq analysis in an OGR1-knockout model [18]. Their data showed that OGR1 knockout resulted in the upregulation of 7 genes and the downregulation of 19 genes in the ipsilateral tMCAO region compared with wild-type (WT) mice. Gene Ontology analysis showed that these downregulated genes were significantly clustered to haptoglobin binding, hemoglobin complex, oxygen binding, and antioxidant activities, all of which are closely associated with neuroprotection against oxidative stress injury. Notably, OGR1 deletion did not affect the expression of other proton-sensitive receptors/channels (*e.g*., GPR4, ASIC1a, TDAG8, PAC) in the brain, either at baseline or after cerebral ischemia. Furthermore, Sun *et al.* have revealed that OGR1 exerts neuroprotection *via* another mechanism: enhancing the Protein kinase R-like ER Kinase (PERK)-eIF2α pathway [15]. PERK is a critical regulator of protein synthesis in stress responses, rapidly activated during cerebral ischemia, and then promotes the dephosphorylation of eIF2α, causing the imbalance of protein synthesis homeostasis [36-38]. Their results showed that OGR1 deletion decreased the expression of PERK and accelerated eIF2α dephosphorylation (approximately 3 hours after tMCAO), while OGR1 activation by Ogerin increased PERK levels and enhanced phosphorylation to sustain translational suppression. Interestingly, it found that Ogerin stimulated eIF2α phosphorylation at 3-6 hours but had little effect at 24 hours, which matches the time window of misfolded protein accumulation [15]. Additionally, another study found that OGR1 mitigated cerebral ischemia-reperfusion injury by negatively regulating NF-κB/HIF-1α [17]. These findings expand the understanding of the role of OGR1 in the brain and offer a promising target for the treatment of ischemic stroke. Although OGR1 has strong neuroprotective effects, its role in long-term ischemic stroke repair remains unclear and requires further investigation.

#### 3.1.2 The Roles of OGR1 in Mechanoreception

 OGR1 is regarded not only as an acid receptor but also as a mechanoreceptor that is very sensitive to changes in blood flow. After cerebral ischemia, vascular resistance increases, blood flow slows, and shear force increases [34], which may be key factors in endothelial cell injury. Moreover, ineffective reperfusion following cerebral ischemia may lead to abnormal hemodynamic changes. It is reasonable to speculate that these changes may affect the function of cerebral vascular Endothelial Cells (ECs) by activating OGR1 and sensing changes in hemodynamics, thereby affecting the recovery process after cerebral ischemia. Therefore, from a vascular perspective, OGR1 may play a role in cerebral ischemia. However, this remains unexplored.

### 3.2 TDAG8

 TDAG8 is a proton-sensitive G-protein-coupled receptor expressed in the Central Nervous System (CNS) and peripheral lymphoid organs [39, 40], which was initially discovered in T lymphocytes. Thus, it is also known as “T cell death-associated gene 8” [41]. TDAG8 is primarily expressed in microglia within the CNS. Additionally, some evidence has shown that TDAG8 is expressed in other cellular types such as astrocytes [42], oligodendrocytes [43], and neurons [44]. It is believed that proton-sensitive GPCRs are distributed on the cell membrane and sense extracellular H^+^ signals to activate internal pathways. A recent study revealed that TDAG8 is also localized in endosomes and stimulates the production of cAMP by sensing H^+^ in endosomes [45]. Importantly, TDAG8 is constitutively active in endosomes, activating cAMP signals regardless of the extracellular pH. However, the physiological and pathological significance of TDAG8 localization in endosomes remains unclear.

#### 3.2.1 The Roles of TDAG8 in the Acute Stage of Ischemic Stroke

TDAG8 has received more attention in the context of cerebral ischemia in recent years, compared to GPR4 and OGR1. In the rat MCAO model, the expression of TDAG8 is significantly upregulated 6 hours after reperfusion. In their study, infarct volume, pro-inflammatory factor production, and neuronal apoptosis were significantly reduced during the acute phase following MCAO by injecting the TDAG8-specific agonist BTB09089 into the lateral ventricle 6 hours before ischemia, and these effects were reversed when TDAG8 was knocked down. In addition, activation of the Akt/cAMP-CREB signaling pathway participates in TDAG8-induced neuroprotection [46]. Similar trends have also been observed in OGD-induced primary cultured cortical neurons, implying that neuronal TDAG8 may be involved in neuroprotection following ischemic stroke injury [44]. Additionally, microglial TDAG8 is considered a crucial target in cerebral ischemia. Sato *et al.* found that TDAG8 was elevated in microglia after tMCAO, while TDAG8 knockout significantly decreased the infarct volume and inflammatory activation of microglia 24 hours after reperfusion [21]. Besides, in TDAG8^-/-^ mice-derived cultured microglia, Jin *et al.* found that acid-activated TDAG8 decreased LPS-induced IL-1β production partly through TDAG8/cAMP/Protein Kinase A (PKA), and then inhibited signal-related kinase and c-Jun N-terminal kinase activities [46]. These studies suggest that TDAG8, located in neurons and microglia, exerts neuroprotective effects during the acute stage of ischemic stroke.

#### 3.2.2 The Roles of TDAG8 in the Sub-Acute Stage of Ischemic Stroke

 Recently, a novel intervention strategy called Delayed Chronic Acidic Postconditioning (DCAPC) has been introduced, aiming to promote neural functional recovery during the subacute phase of stroke [20]. Using a mouse photothrombotic model of stroke, this strategy involves brief exposure of mice to 5%, 10%, and 20% CO_2_ daily to mildly and transiently lower brain pH. This approach significantly promotes the recovery of motor function, alleviates the overactivation of astrocytes and microglia, and enhances the expression of markers of axon growth and synaptogenesis. More importantly, this intervention strategy offers a wide therapeutic time window of at least seven days, providing a new approach for the development of more effective stroke rehabilitation treatments. Interestingly, the present study revealed a persistent increase in TDAG8 levels during the subacute phase. By utilizing TDAG8^-/-^ mice and administering an Adeno-Associated Virus (AAVs) designed to overexpress TDAG8 in the peri-infarct cortex of TDAG8^-/-^ mice, it was further discovered that brain-derived TDAG8 is a direct target of DCAPC, which elicits neuro-reparative effects.

 In another study, it was found that BTB09089, a TDAG8-specific agonist, significantly improved motor functional recovery, synaptic plasticity, and inhibited glial overactivation and glial scar progression following photothrombotic injury in mice with a time window comparable to that of DCAPC [13]. Nevertheless, these effects were not observed in TDAG8^-/-^ mice. Overexpression of TDAG8 specifically in cortical neurons of the peri-infarct area in TDAG8^-/-^ mice restored the therapeutic effects of BTB09089, which were related to the elevation of phosphorylated CREB. The overexpression of neuronal TDAG8 further extended the therapeutic window of BTB09089 to 14 days post-injury. This suggests that activating neuronal TDAG8 can improve neurological function recovery and brain tissue repair after cerebral ischemia through delayed BTB09089 treatment. Thus, overexpression of neuronal TDAG8 may be a potential strategy for expanding the time window of TDAG8 agonist treatment for neural repair after cerebral ischemia [13]. In a traumatic brain injury mouse model, which exhibits processes like those of ischemic stroke, DCAPC exhibited therapeutic effects comparable to those seen in the ischemic stroke model [47]. Furthermore, using AAVs to selectively overexpress astrocytic TDAG8, it was found that astrocytic TDAG8 activation had a critical effect on neuro-restoration following controlled cortical impact, which exhibited upregulated BDNF, promoted motor function recovery, reduced infarct volume, and overactivation of astrocytes and microglia [14]. In addition, as with previous results, the overexpression of astrocytic TDAG8 extended the therapeutic time window for DCAPC. These findings highlight the potential of TDAG8 as a crucial target for neural repair in patients with ischemic stroke. Collectively, these findings emphasize the significance of TDAG8 in brain tissue repair during the subacute phase following acute brain injury. These studies provide compelling evidence for the development of effective targets and drugs against ischemic stroke, underscoring the potential of TDAG8-centric interventions for facilitating functional recovery and tissue repair after ischemic stroke. In the future, the role of TDAG8 derived from different cell types in the recovery process after cerebral ischemia, as well as the detailed molecular mechanisms and cell-cell interactions it mediates, still awaits in-depth investigation.

### 3.3 GPR4

 GPR4 is expressed in various tissues throughout the body [48]. Notably, RNA-sequence data and immunofluorescence revealed that GPR4 is largely expressed in cerebral ECs [49], and single-cell PCR detected GPR4 in multiple brain neuronal groups, including the retrotrapezoid nucleus, catecholaminergic area, and raphe nuclei [50]. Hosford *et al*. detected GPR4 in lateral septum neurons using RNA-scope FISH (Fluorescence *In Situ* Hybridization), a method that revealed a newly identified neuronal group expressing GPR4 [50]. Additionally, GPR4 is expressed in glial cells, albeit at low levels. These findings underscore the diverse expression of GPR4 in the CNS, implying its multiple functional roles in the brain.

#### 3.3.1 The Roles of GPR4 in Cerebral Ischemia

To date, research on the role of GPR4 in cerebral ischemia is limited. The expression levels of GPR4 are upregulated during the acute period in MCAO and photothrombotic ischemia models, suggesting that GPR4 plays a role in acute cerebral ischemia [18]. Surprisingly, no significant changes in infarct volume were observed after systemic GPR4 knockout in mice [21]. However, a recent study found that inhibiting GPR4 one hour after the subarachnoid hemorrhage in mice markedly reduced neuronal ferroptosis, oxidative stress injury, and neurobehavioral dysfunction, thereby exhibiting neuroprotective effects [51]. In other ischemia-related models, notable effects of GPR4 have been observed. In murine models of acute hind limb ischemia-reperfusion injury, GPR4 activation elicits tissue edema, promotes the formation of inflammatory exudates, enhances the expression of endothelial cell adhesion molecules, and augments leukocyte infiltration. This inflammatory response is significantly attenuated when GPR4 is genetically ablated or pharmacologically blocked using NE 52-QQ57 [52]. Meanwhile, a recent study showed that the expression of GPR4 was elevated in a renal ischemia-reperfusion injury model, and the downregulation of GPR4 inhibited cell apoptosis and damage by decreasing the nuclear transcription factor C/EBP-Homologous Protein (CHOP) [53]. Based on these findings, it cannot be confirmed whether the role of GPR4 in cerebral ischemia is negligible. Therefore, further validation through conditional knockout experiments and pharmacological interventions is required to disrupt the expression and activity of GPR4 across different cellular subtypes and phases following ischemic stroke.

#### 3.3.2 The Roles of GPR4 in Brain Microvascular Endothelial Cells (BMECs)

 GPR4 plays a key role in regulating cerebrovascular reactivity and inflammation in BMECs. A recent study found that in the cortex and amygdala, CO_2_/H^+^ promoted cerebrovascular dilation, which was dependent on endothelial GPR4/ Gα_q/11_ signaling [54]. Conversely, in the brainstem, CO_2_/H^+^ facilitated vasoconstriction. This discrepancy is attributed to unique gene expression patterns and the release of distinct vasoactive factors in different brain regions. In addition, Liu *et al.* found that Lysophosphatidylcholine (LPC) significantly upregulated GPR4 expression and the levels of inflammatory factors in BMECs, and knockdown of GPR4 attenuated the effects of LPC, indicating that GPR4 is involved in the inflammatory responses of BMECs [55].

In other ECs, activation of the GPR4 receptor promotes the release of endothelial proinflammatory factors, accelerating EC injury [56-58]. Chen *et al.* have found that acidosis-induced GPR4 activation increases the adhesion of primary Human Umbilical Vein Endothelial Cells (HUVECs) *via* the cAMP/EPAC pathway [58]. Dong *et al.* have found that the NF-κB pathway is critical for GPR4-induced inflammatory gene expression by comparing the HUVECs' global gene expression of acidosis response [59]. Besides, GPR4 has also been implicated in neovascularization *via* the Notch or STAT3/VEGF signaling pathways in human ECs or endothelial progenitor cells [60, 61]. Additionally, it has been reported that the elevation of GPR4 promotes vascular formation in EC through activating the SP1/VEGF-A pathway [62]. Despite these advancements, the precise role of GPR4 expression in BMECs in regulating cerebrovascular activity, endothelial adhesion, inflammation, and neovascularization after ischemic stroke requires further exploration.

#### 3.3.3 The Roles of GPR4 in Neuronal Apoptosis

 GPR4 exerts a role in neuronal apoptosis. In an *in vitro* model of cerebral ischemia, GPR4 and its downstream regulator, CHOP, were significantly increased following Oxygen-Glucose Deprivation/Reoxygenation (OGD/R) in SH-SY5Y cells (a neuron-like cell line) [22], while a GPR4 antagonist (NE 52-QQ57) reversed OGD/R-induced cellular apoptosis and LDH release. Furthermore, overexpression reversed these effects, suggesting that GPR4 promotes neuronal apoptosis *via* CHOP signaling. In addition, in 1-methyl-4-phenyl-1,2,3,6-tetrahydropyridine-induced Parkinson’s disease mice, inhibiting expression of GPR4 protected dopaminergic neurons from oxidative stress injury and caspase3-dependent apoptosis [63]. Considering the heterogeneity of GPR4 expression across different neural nuclei, GPR4 has emerged as a noteworthy neuroprotective intervention target for cerebral ischemic events that occur in neural nuclei with GPR4 expression, warranting further research.

## 4 ASIC1a

 ASICs are proton-gated cation channels that belong to the degenerin/epithelial sodium channel superfamily and are distributed throughout both the central and peripheral nervous systems [64]. In mammals, six ASIC subtypes (ASIC1a, ASIC1b, ASIC2a, ASIC2b, ASIC3, and ASIC4) have been identified up to now [65]. ASICs have a unique structure with two transmembrane domains and a pore region shaped like a clasped hand [66, 67]. Physiologically, the pores are closed, but start to open at a low pH [67, 68]. Within the ASICs family, ASIC1a has received the most attention in the context of ischemic stroke.

ASIC1a is a proton-gated ion channel involved in calcium ion (Ca^2+^) transport. ASIC1a exhibits the highest sensitivity to pH among the ASICs, being activated within a pH range of 7.0 to 4.0 and demonstrating peak sensitivity at a pH of 6.9 [69, 70]. ASIC1a is widely distributed in the hippocampus, cortex, amygdala, and striatum regions of the brain and is mainly located in neurons and glia. In neurons, ASIC1a is mainly expressed on the soma and dendritic spines [71], whereas in glia, it is mainly expressed on the surface. Specifically, ASIC1a was expressed in the cytoplasm and nuclei of astrocytes [71]. When ASIC1a is activated, the current amplitude increases with the elevation of extracellular H^+^ concentration, yielding half-maximal activation at pH ~6.5 [72]. As the extracellular H^+^ concentration continues to rise, ASIC1a's current amplitude trends towards saturation and then decreases due to increased proton stimulation, leading to rapid desensitization [69, 73]. This process usually occurs within hundreds of milliseconds to several seconds in mammals [74, 75]. Desensitization protects neurons from sustained excitation injury during acidosis (*e.g*., tissue ischemia and inflammation), aiding in synaptic transition and plasticity [76]. The speed and degree of ASIC1a desensitization depend on pH, proton stimulation duration, recovery time, and Ca^2+^ permeability [69, 77, 78]. However, the intricate relationships and underlying mechanisms remain largely undefined, which is important for exploring novel intervention strategies for acid-related CNS diseases such as cerebral ischemia.

### 4.1 The Roles of ASIC1a in Ischemic Stroke

A series of studies have shown that inhibiting ASIC1a significantly reduces the volume of infarction and neuronal damage and improves motor function in MCAO models [23, 79]. Significant progress has been made in developing therapeutic drugs targeting ASIC1a. PcTx1, Amiloride, A-317567, ApeTx2, and nafamostat are common inhibitors of ASIC1a [80, 81]. Besides, Qiang *et al*. developed a combinatorial functional antibody, ASC06-IgG1, against human ASIC1a by using nanodisc technology [25]. This functional antibody selectively blocks the ASIC1a channel using a mechanism that is noncompetitive with respect to proton concentration, showing strong neuroprotective effects against ischemic stroke injury.

 Cerebral ischemia-induced extracellular acidification promotes the activation of ASIC1a, which leads to Ca^2+^ influx, causing intracellular Ca^2+^ overload and triggering multiple intracellular signaling pathways, including the activation of calcium-dependent enzymes and the production of free radicals, exacerbating neuronal injury and accelerating cellular death [82-84]. Besides, it has been found that activated ASIC1a by H^+^ specifically binds to the intracellular death-inducing kinase (RIP1) during cerebral ischemia. This interaction triggers the phosphorylation of RIP1, which in turn activates necroptosis in neurons. Importantly, inhibiting RIP1 phosphorylation or knocking down its expression can effectively suppress programmed necrosis in neurons [26]. Other new mechanisms of ASIC1a have been discovered in recent years, including the recognition and activation of ASIC1a by glutamate, mitochondrial energy metabolism, and neurogenesis. These findings suggest that ASIC1a plays a pivotal role in ischemic stroke through other mechanisms.

### 4.2 Positive Allosteric Modulatory Roles of Glutamate for ASIC1a in Ischemic Stroke

 Glutamate, as the first messenger, is traditionally recognized to activate NMDAR (N-methyl-d-aspartate receptor), then contributing to neuronal death in stroke [85], while H^+^ activates ASIC1a, potentially occurring simultaneously. These two channels may interact through intracellular signaling pathways, leading to an exacerbation of neuronal death [76]. Recently, Lai *et al.* reported that glutamate serves as a positive allosteric modulator for ASIC1a, exacerbating neurotoxicity. Specifically, glutamate enhances the affinity of ASIC1a for protons and elevates their open probability, ultimately resulting in Ca^2+^ overload and exacerbating ischemic neurotoxicity in both *in vitro* and *in vivo* models. Furthermore, through the application of computer simulation techniques and high-throughput molecular screening, researchers have successfully identified a glutamate-binding pocket located within the extracellular domain of ASIC1a. They also identified a small-molecule compound, LK-2, which competes with glutamate for this binding pocket, thereby preventing glutamate from binding to ASICs and mitigating ischemic brain injury [16]. LK-2 demonstrated ideal neuroprotective effects, reducing cerebral infarction volume by approximately 53.6% and improving motor function and prognosis in the tMCAO model of mice. This study established a conceptual paradigm for understanding the mechanisms of neurotoxicity in ischemic stroke and for developing new stroke treatment strategies that target the glutamate-binding site on ASIC1a, independent of NMDARs.

### 4.3 Roles of ASIC1a in Mitochondrial Energy Metabolism

It is well known that mitochondrial energy metabolism disorder is involved in the pathological process of ischemic brain injury. The activation of ASIC1a leads to Ca^2+^ overload, which imposes a burden on the mitochondria, prompting them to enhance energy production to maintain intracellular calcium ion homeostasis [82-84]. This high energy demand can exert strain on mitochondrial function, particularly when the energy supply is constrained. Under cerebral ischemia, the oxidative phosphorylation of mitochondria is impaired, resulting in reduced ATP production. Thus, the activation of ASIC1a causes oxidative injury to the mitochondria. Recently, Savic *et al.* found that ASIC1a activation may regulate neuronal survival by affecting mitochondrial membrane permeability transition, thereby increasing the release of cytochrome c and activating apoptotic pathways [86]. Besides, they also found that in cultured primary cortical neurons from mice, a physiologically relevant decrease in pH 7.0 triggers ASIC1a-dependent cytosolic Na^+^ and Ca^2+^ waves that propagate to mitochondria. Inhibition of ASIC1a through the specific inhibitor PcTX1 or ASIC1a gene knockout blocked the mitochondrial Na^+^ and Ca^2+^ signals. Importantly, physiologically activating ASIC1a by shifting the pH to 7.0 enhances basal respiration, maximal respiration, and ATP-coupled respiration [86]. This suggests that ASIC1a is crucial for linking physiologically mild changes in the extracellular pH to mitochondrial ion signaling and metabolic activity.

Mitochondria serve as cellular "energy factories," essential for neuronal survival. ASIC1a activation can alter mitochondrial shape and function, thereby affecting energy supply and neuronal survival, possibly by affecting mitochondrial membrane permeability. Although ASIC1a is a promising target, its association with mitochondrial changes during cerebral ischemia remains unclear. Further research is essential to elucidate these mechanisms, as they may hold the key to developing novel therapeutic strategies that preserve mitochondrial health and neuronal function during ischemic insults.

### 4.4 Role of ASIC1a in Neurogenesis

Stimulation of Neural Progenitor Cell (NPCs) proliferation and differentiation is an important strategy for promoting neurological recovery following cerebral ischemia. Recently, researchers discovered that knocking out ASIC1a promotes NPC migration and neurogenesis in the ischemic penumbra, thereby enhancing behavioral recovery after stroke [24]. This suggests that ASIC1a is a novel target for neural regeneration in ischemic stroke. Therefore, ASIC1a not only serves as a neuroprotective target but also has potential as a therapeutic strategy for neural repair, offering a promising role in the treatment of ischemic stroke.

### 4.5 Roles of ASIC1a in Synaptic Plasticity

 ASIC1a is considered a pivotal target in regulating synaptic plasticity over the long term [87-91]. Zha *et al.* utilized combined Ca^2+^ imaging and electrophysiological techniques to demonstrate that hippocampal neuronal ASIC1a is localized primarily in the postsynapse, and its activation causes Ca^2+^ influx, thereby affecting postsynaptic function. Deletion of ASIC1a reduced dendrite spine density, while overexpression increased density [91]. Furthermore, Yu *et al.* demonstrated that ASIC1a is essential for motor functions and programmed learning by regulating synaptic plasticity of spinous neurons in the striatum. Their research showed that ASIC1a knockout increased dendritic spine density while impairing spine morphology and postsynaptic architecture, concomitant with decreased NMDA receptor dysfunction. These changes induced by ASIC1a depletion are mediated by reduced phosphorylation of Ca^2+^/calmodulin-dependent protein kinase II and extracellular signal-regulated protein kinase, revealing that ASIC1a promotes striatum-associated procedural learning and memory of excitatory synaptic function [90]. However, it remains unclear whether ASIC1a-mediated synaptic plasticity is involved in rehabilitation following ischemic stroke. Bridging this knowledge gap could unveil novel neurorestorative strategies for stroke rehabilitation. Notably, it has developed an astrocyte-targeted optogenetic approach using adenoviral ArchT expression that enables precise ASIC1a activation *via* light-induced local acidification in awake animals and brain slices [92]. This approach offers possibilities for precise control of ASIC1 activation to enhance synaptic plasticity following ischemic stroke.

## 5 PAC

 PAC is known as a proton-activated Chloride Ion (Cl^-^) channel and is present in many mammalian cells [93]. The pH sensitivity of the PAC was lower than that of the ASIC1a channel. It is activated at ~pH 5.5 at room temperature [5], whereas PAC initiates its opening at roughly pH 6.0 at 37^o^C [94], thereby indicating its temperature-sensitive characteristic. Therefore, the activation of the PAC channel requires severe acidosis. Due to its outward rectifying properties, PAC is also called “acid-sensitive outwardly rectifying anion channel” [95]. Generally, PACs are believed to contain two transmembrane domains, intracellular N-termini and C-termini, and an extracellular domain [27, 96]. According to the previous studies, PAC is expressed in multiple organs, including the brain, bone marrow, kidney, spleen, lung, *etc*. [27, 97]. In the brain, this channel is mainly expressed on neurons, microglia, and astrocytes [95, 97].

### 5.1 Roles of PAC in Ischemic Stroke

It has been proposed that PAC often functions synergistically with other ion channels involved in ischemic stroke. Under the stimulation of extracellular acidification, ASIC1a and transient receptor potential melastatin channels are activated, leading to a significant influx of Ca^2+^ and Mg^2+^, which then promotes PAC-mediated Cl^-^ entry [98]. In contrast, continuous extracellular acidification also induced high H^+^ influx, accelerating the operation of Na^+^-H^+^ exchanger 1, and an increasing amount of Na^+^ entered the cells to exchange for H^+^. Na^+^ accumulation activates the Na^+^-Ca^2+^ exchanger, promoting Ca^2+^ influx, which further triggers a series of cellular cascade reactions and aggravates injury [98, 99].

In a permanent MCAO mouse model, the activity of PAC was significantly enhanced. PAC knockdown reduces acidosis-induced neuronal death and infarct volume [27]. These results support the notion that the activation of PAC channels and subsequent chloride entry into neurons play a key role in acid toxicity-related brain injury. Additionally, low-temperature therapy is believed to be one of the most reliable and efficient strategies for preventing ischemic stroke from excitotoxicity and brain injury, which mainly exerts protective effects by decreasing the ratio of cerebral metabolism [97]. The temperature-sensitive properties of PAC may be one of the underlying mechanisms of low-temperature therapy [100]. It was reported that the precise temperature reduction of the acidifying brain tissue by therapeutic hypothermia can increase the PAC opening threshold, thereby closing the channel, reducing Cl^−^ influx, improving cytotoxic edema, and achieving neuroprotection [100].

### 5.2 Roles of PAC in Endosomal pH and Endocytosis

Recently, Osei-Owusu *et al*. found that PAC is transferred from a newly identified plasma membrane channel to the endosome, where it forms an endoplasmic reticulum organ Cl^-^ channel [94]. Their findings revealed that PAC regulates the endosomal pH and chloride levels by modulating the chloride current in the endosome, which ultimately influences transferrin receptor-mediated endocytosis. Specifically, in the absence of PAC, endosomal chloride concentrations increased, leading to a decrease in pH and enhancement of transferrin receptor-mediated endocytosis. Conversely, PAC overexpression decreased endosomal pH and reduced transferrin uptake. Therefore, PAC serves as a low-pH sensor within endosomes, mitigating excessive acidification by releasing chloride ions from the internal cavity [101]. Therefore, PAC channels are important for maintaining endosomal pH homeostasis and regulating receptor-mediated transferrin endocytosis. Given that ferroptosis following cerebral ischemia constitutes a pivotal mechanism underlying neuronal death and that the accumulation of transferrin can intensify this process, it is reasonable to speculate that PAC localized in endosomes may also affect neuronal ferroptosis induced by cerebral ischemia, which needs further investigation. A recent study reported that PAC, located at the early and recycling endosomes of neurons, plays an important role in inhibiting endosomal hyperacidification and thereby enhances AMPAR endocytosis during long-term depression. This study confirmed that PAC is a key target for synaptic plasticity through regulating endosomal pH balance in neurons [102]. Given the importance of synaptic remodeling in functional recovery following cerebral ischemia, it is worthwhile to further explore the roles of PAC-mediated endosomal pH in post-ischemic synaptic plasticity, which may reveal novel therapeutic strategies for stroke rehabilitation.

## 6 CLINICAL POTENTIAL

In recent years, a series of drugs targeting proton-sensing receptors have been developed, and some have been applied in ischemic stroke models. For example, ASC06-IgG1, a combinatorial functional antibody against human ASIC1a, can produce a robust neuroprotection against cerebral ischemic reperfusion injury. In mice, it has a wide therapeutic window of up to 3 hours, suggesting its clinical prospects [25]. LK2, a novel small-molecule ASIC1a inhibitor targeting the glutamate-ASIC1a interaction site, can not only attenuate ischemic brain injury but also avoid the side effects associated with traditional NMDA receptor antagonists [16]. In addition, BTB09089, the specific agonist of TDAG8, is reported to be applied in the acute or sub-acute phases of ischemic stroke, exhibiting double beneficial effects on alleviating ischemic brain injury and promoting neurological function recovery. However, the clinical translation of these drugs targeting these receptors/channels faces multiple challenges. Currently, the majority of data on these drugs are derived from rodent animals or cell models, necessitating further preclinical studies in other species, especially safety evaluations.

In addition to therapeutic drugs targeting proton-sensing receptors/channels, several neuroprotective strategies have been proposed to mitigate ischemic brain injury. For example, inhalation of CO_2_ not only has neuroprotective effects in the acute phase of cerebral ischemia but also promotes neural repair in the subacute phase. Notably, it has been established that 5-8% CO_2_ has been used in clinical practice, such as 5% CO_2_ to inhibit seizures in children [103] and 8% CO_2_ to evaluate cerebrovascular reactivity [104], both of which have shown great safety and tolerance. Meanwhile, animal experiments have verified the efficacy of 5-10% CO_2_ in ischemic brain injury, suggesting that this strategy has great translational potential in ischemic stroke [9, 14, 47]. In addition, gene therapy using cell-type-specific AAV vectors provides a way to precisely regulate the expression of proton-sensing receptors/channels following cerebral ischemia with safety and targeting ability [105].

## 7 LIMITATIONS AND FUTURE DIRECTIONS

 As shown in Table **1**, most interventions were verified in only one type of animal model of ischemic stroke. This limited validation raises concerns regarding the broader applicability and efficacy of these interventions across diverse stroke conditions. Moreover, current studies predominantly utilize healthy and young animal models, but clinical stroke patients typically present with multiple comorbidities (*e.g*., diabetes, obesity, and cardiovascular diseases), aging, and polypharmacy regimens. Further research encompassing multiple models may provide more comprehensive insights into their therapeutic potential. 

Meanwhile, the roles and mechanisms of proton-sensing receptors/channels in ischemic stroke have not yet been fully clarified. Future studies should further delineate the roles played by these receptors at different stages of cerebral ischemia, particularly their mechanisms in the subacute and chronic phases, and their influence on neural repair in response to local microenvironment acidification. Furthermore, the specific roles that proton-sensing receptors/channels play within the intricate network of interactions among various cell types within the neurovascular unit following cerebral ischemia deserve thorough investigation. Notably, the distribution of some receptors (*i.e*., GPR4) exhibits significant brain region specificity, potentially related to differences in cell subpopulations across different brain regions. The integration of single-cell sequencing with spatial transcriptomics will facilitate a more precise analysis of the expression, distribution, and functional properties of these receptors/ channels, with a particular focus on their spatiotemporal changes after cerebral ischemia, thereby providing a theoretical foundation for precision medical intervention strategies. Moreover, certain proton-sensing receptors, such as GPR4 and GPR65, are also expressed in peripheral immune cells that play a pivotal role in the neuroinflammatory response following ischemic brain injury, examining the distinct effects of these receptors on neuroinflammation from the perspective of peripheral immune cells may further enrich our understanding of their roles in ischemic brain injury. Additionally, recent studies have also revealed the unique distributions and functions of GPR65 and PAC on endosomes, prompting consideration of similar distributions and functions for other proton-sensitive receptors/channels, which offers a new perspective for understanding the mechanisms of these targets in the pathophysiological processes of cerebral ischemia.

## CONCLUSION

In summary, proton-sensitive receptors/channels play pivotal roles in the pathophysiological processes associated with ischemic stroke. They exhibit distinct mechanisms in response to the pH reduction caused by cerebral ischemia, thereby regulating neuronal damage and repair, glial cell activation, and cerebrovascular function. As the comprehension of these receptors/channels becomes increasingly clarified and thoroughly explored, therapeutic drugs or strategies targeting proton-sensitive receptors/channels will exhibit broad clinical translation prospects, opening new avenues for the treatment of ischemic stroke.

## Figures and Tables

**Table 1:** The main studies of proton-sensitive receptors/channels in the context of cerebral ischemia in the last ten years.

**Receptors/ Channels**	**Main Studies**	**Ischemic Models**	I**nterventions Targeting to Proton Receptors/Channels**	**Findings**
OGR1	[15]	tMCAO in WT and OGR1^-/-^ mice	Ogerin, administered immediately, 3 hours and 5 hours respectively after reperfusion	OGR1 exerts neuroprotection by time-dependently enhancing the PERK-eIF2α pathway post-ischemia.
	[17]	MCAO/R in WT; OGD/R in SH-SY5Y and HMC3 cell lines	Ogerin, administered 2 weeks before MCAO/R, once a week	OGR1 activation exerts neuroprotective effects against cerebral ischemia-reperfusion injury *via* the NF-κB/Hif-1α pathway.
	[18]	tMCAO in WT and OGR1^-/-^ mice	None	RNA sequencing analysis shows OGR1-regulated genes such as H2-Aa, Ppbp, Siglece, and Tagln.
	[19]	tMCAO in WT and OGR1^-/-^ mice OGD in organotypic cortical slices from WT and OGR1^-/-^ mice	AAV-CMV-OGR1	OGR1 plays a neuroprotective role in cerebral ischemia through PKC pathway.
TDAG8	[13]	PTI in WT and TDAG8^-/-^ mice	BTB09089, administered at days 3, 7, or 14 post-injury AAV-Syn-TDAG8, administered 2 weeks before PTI	Delayed BTB09089 treatment promotes the neurorestoration through targeting neuronal TDAG8 following cerebral ischemia.
-	[20]	PTI in WT and TDAG8^-/-^mice	5%/10%/20% CO_2_ inhalation administered at days 3-7, 7-21, or 3-21 post-injury AAV-CMV-TDAG8, administered 3 weeks before PTI	Delayed chronic acidic postconditioning improves motor function recovery and brain tissue repair after ischemic stroke by activating TDAG8.
	[21]	tMCAO in WT and TDAG8^-/-^ mice	None	TDAG8 plays neuroprotective effects against the acute ischemic reperfusion injury.
GPR4	[22]	OGD/R in SH-SY5Y cells	shRNAs for GPR4 silencing	GPR4 inhibition attenuates OGD/R-induced injury in SH-SY5Y cells by suppressing CHOP-mediated apoptosis.
ASIC1a	[16]	tMCAO in WT ASIC1a^-/-^ mice; OGD/R in primary cortical neurons from WT and ASIC1a^-/-^ mice	LK-2, administered 30 min after tMCAO/R	Glutamate exacerbates ischemic brain injury through positive allosteric regulation of ASIC1a.
	[23]	tMCAO in WT and ASIC1a^-/-^ mice	PcTX1, administered 30 min after tMCAO	Both genetic knock-out and acute pharmacological inhibition of ASIC1a improve long-term behavioral recovery after ischemic stroke.
	[24]	dMCAO in WT and ASIC1a^-/-^ mice	PcTX1/Amiloride, administered 30 minutes before dMCAO AAV-CMV-ASIC1a, administered 2 weeks before dMCAO	ASIC1a inhibits neural progenitor cell migration and neurogenesis in ischemic stroke.
	[25]	tMCAO in WT mice	ASC06/PcTX1, administered 3 hours after tMACO	It has found that ASC06, a combinatorial functional antibody, protects cell death from ischemic stroke.
	[26]	tMCAO in WT mice	None	Tissue acidosis induces neuronal necroptosis *via* activating ASIC1a mediated RIP1 during cerebral ischemia.
PAC	[27]	pMCAO in WT and Pac^-/-^ mice	None	PAC (TMEM206) is a proton-activated chloride channel (PAC), plays a key role in acidosis related cell death and ischemic brain injury.

**Abbreviations**: dMCAO, distal middle cerebral artery occlusion; OGD/R, oxygen-glucose deprivation/reoxygenation; pMCAO, permanent middle cerebral artery occlusion; PTI, photothrombotic ischemia. tMCAO, transient middle cerebral artery occlusion.

## LIST OF ABBREVIATIONS

AAVs: Adeno-associated Virus
ASICs: Acid-sensing Ion Channels
BDNF: Brain-derived Neurotrophic Factor
BMECs: Brain Microvascular Endothelial Cells
cAMP: Cyclic Adenosine Monophosphate
CHOP: C/EBP-Homologous Protein
CNS: Central Nervous System
CREB: cAMP-responsive Element-binding Protein
DCAPC: Delayed Chronic Acidic Postconditioning
ECs: Endothelial Cells
ER: Endoplasmic Reticulum
GPCRs: G Protein-coupled Receptors
LPC: Lysophosphatidylcholine
LTD: Long-term Depression
MCAO: Middle Cerebral Artery Occlusion
NPC: Neural Progenitor Cells
OGD/R: Oxygen-glucose Deprivation/Reoxygenation
OGR1: Ovarian Cancer G Protein-coupled Receptor 1
PAC: Proton-activated Chloride Channel
PARK2: Parkinson's Disease (Autosomal Recessive Juvenile) 2 Parkin
PKA: Protein Kinase A
PKC: Protein Kinase C
RIP1: Receptor-interacting Protein Kinase 1
TDAG8: T Cell Death-associated Gene 8
